# When Academic Freedom Becomes Dangerous: Nursing in an Age of Censorship

**DOI:** 10.1111/nin.70044

**Published:** 2025-06-26

**Authors:** Elisabeth Dahlborg, Ivan Andrés Castillo, Ellinor Tengelin

**Affiliations:** ^1^ Department of Health Sciences University West Trollhättan Sweden; ^2^ Department of Health Sciences, Occupational Health Science Mid‐Sweden University Östersund Sweden

We, together with many in the nursing community, look with fear and dismay at the reactionary forces that seek to hinder free research. For almost 15 years, our research group has been working in the tradition of emancipatory nursing (Kagan et al. 2010) by applying a norm‐critical approach in research and education to address healthcare inequity (Castillo et al. 2024; Tengelin et al. 2020). We conduct norm‐critical research and education in the field of caring and nursing sciences, an area aimed at promoting and preserving people's health and well‐being. Our fundamental standpoint is that healthcare should be characterized by inclusion, equal value for all, fairness, and participation. Our research group studies the mechanisms behind exclusion in healthcare and contextualizes them from a societal standpoint. Harmful inequities within society are becoming undeniable, especially as economic living conditions grow more unequal, both within and between countries. Even in a welfare state like Sweden, certain groups experience poorer health, shorter life expectancy, and less access to care and treatment than other groups, for example, women with on or little educational background (Public Health Agency of Sweden 2025). As three Canadian nurses emphasize (Goldberg et al. 2025), *Diversity, Equity, and Inclusion* (DEI) is a broad and inclusive concept aimed at counteracting continued discrimination against marginalized groups. DEI seeks to strengthen the rights of communities that have been denied equity, such as Black individuals, Indigenous peoples, women, 2SLGBTQ+ individuals, people of color, and those with disability. One reason for marginalization are the norms that prevail in society, norms related to gender, sexuality, race, and ethnicity. These norms also include those that marginalize certain age groups, people with disabilities, and those whose social class does not align with the dominating middle class (Kalonaityté 2014; Nye et al. 2023). By highlighting norms as an issue that healthcare professionals and nurses actively must acknowledge and criticize in developing strategies for a more inclusive healthcare system, our aim is and has been to contribute to fairer and more sustainable healthcare for all.

## 1 Pushback Against Academic Freedom

Over the past decade, academic freedom has been decreasing globally, not just in autocracies and emerging democracies but also in longstanding liberal democracies such as the US and Great Britain (Lott 2024). This intrusion on academic freedom and research can be exemplified by the backdrop of the politicization of reproductive health and reproductive healthcare services in the United States. Thus, the current state of academic freedom is under serious threat, and if the restrictions and bans announced by the current US administration gain international traction, much of the research we conduct could become impossible.

Editing and removal of work and documents in the United States has been underway since the Office of Personnel Management issued orders ending programs that use taxpayer money to promote or reflect the so called “gender ideology.” For example, in March 2025 researchers at the American Center for Disease control and Prevention (CDC) were instructed to remove reference or terms that included “gender,” “transgender,” “pregnant person/people,” “LGBT,” “non‐binary,” “assigned male/female at birth,” and “biological male or female” (Faust 2025). Because of this prohibition, the CDC removed or edited references to transgender people, gender identity, and equity from its website, a removal that will have implications for researchers who have used this comprehensive data material. In the wake of significant reductions in the National Institutes of Health (NIH) research funding, the administration has dismissed the Director of the National Institute of Nursing Research (NINR) and proposed integrating NINR into a larger entity. NIH serves as the primary agency within the US government dedicated to advancing biomedical and public health research (Georges 2025).

The US government's restriction on research agencies has, moreover, affected scientific journals, which have been encouraged by the government to withdraw manuscripts that do not comply with the executive order on gender ideology. These incursions on research and academic freedom will affect how emancipatory and norm‐critical nursing knowledge is disseminated to the research community, inhibiting continued research.

*Poets, Essayists, Novelists* (PEN) was founded in 1921 with the purpose of protecting the free exchange of ideas and fighting against censorship and the persecution of authors. Today, the organization includes writers of all types and operates in 90 countries. At the end of April 2025, PEN published a list of over 350 “forbidden words” that are not considered acceptable for researchers in the United States, for example, in applications for research grants. Many of these “forbidden words” are central to our field of norm‐critical research.

During 2025, institutions in the United States have been shut down and funding has been revoked. The country is significantly retreating in matters of academic freedom, according to *Academic Freedom Index* (AFI) which assesses de facto levels of academic freedom based on five indicators: freedom to research and teach; freedom of academic exchange and dissemination; institutional autonomy; campus integrity; and freedom of academic and cultural expression (Kinzelbach et al. 2025). A columnist for the Swedish Research Council (Flinkfeldt 2025) draws on the late medieval philosopher Machiavelli, who described the mechanisms of power and emphasized that a leader should prioritize efficiency and power over morality to maintain control. Although written in the 1500s, Machiavelli's idea remains highly relevant today. His view was that people act irrationally, being swayed by emotions, self‐interest, and societal pressures. His assertion that “people are foolish—everyone can see, but few understand” can be applied to how the administration in the United States acts by an Machiavallian ethos in its strategic manipulation of facts, reinforcing the idea that although folly is visible to all, true understanding remains elusive. However, creating and spreading understanding is what researchers dedicate themselves to, and therefore, researchers are dangerous to those who seek absolute power. Thus, controlling universities is a classic strategy for despots (Flinkfeldt 2025). Censorship of texts and research toward this end is a longstanding historical and political phenomenon. Recent developments originating in the United States, though with significant transnational implications, reflect a broader pattern of restricting access to knowledge, evoking parallels with ideologically driven acts such as the 1930s book burnings in Germany, among many historical examples.

## 2 Power Based on Knowledge or Unfounded Opinion

Today, “fake news” is widespread, especially on social media platforms, where discussions about freedom of speech can be distorted. Opinions are often built on misinformation rather than knowledge. According to Goldberg et al. (2025), one of the more alarming actions taken by the US administration is the use of revisionist history, which impacts areas such as education and national art institutions. This practice involves distorting or reinterpreting established historical facts to serve an alternative narrative or agenda.

Aristotle distinguishes between insight (*nous*) and knowledge (*episteme*), where *episteme* is research‐based and leads to a deeper understanding. Arendt (2017) further differentiates *insight*, which stems from philosophical and scientific inquiry, from *opinion*, which develops within civic discourse around politics, economics, and labor. Despite opinions often being devalued, Arendt argues that they, rather than scientific truths, shape the foundations of power. According to the intellectual historian Sven Erik Liedman (2020), opinions often reflect personal perspectives on morality, society, and learning, which do not require extensive knowledge. This distinction is crucial, particularly in equity‐oriented nursing research where reaching a broader audience with scientific insights about mechanisms behind healthcare exclusion is essential for improving health and well‐being. Ethical nursing knowledge is a fundamental value for our practice (American Nurses Association 2025; Carper 1978) and there is an ongoing discussion among nurse leaders about our ethical obligations (Chinn 2025; Fowler 2025). Yet, we sometimes fail in our moral duty, by giving space to arguments that dilute ethical clarity for the sake of appearing “*neutral*,” where neutrality becomes a form of complicity (Thorne 2025). As nurses and academics, we must not remain passive observers. Instead, we have a responsibility to actively reject false equivalences and ensure that our discourse upholds truth and accuracy, rather than merely being presented as a perspective.

## 3 Why a Nursing Reaction Is Necessary

Academic freedom is a fundamental value for higher education and research, a prerequisite for well‐functioning democracies and sustainable societal development. This includes the right to independently develop, acquire, and apply knowledge and ideas through research, without influence from scientifically irrelevant ideological, political, or commercial interests. To understand the motives behind the current censorship, we can turn to the distinction between insight and knowledge. We are deeply concerned about the increasing pressure on academic freedom in Europe and globally, particularly in areas such as health and equality. Political policy changes, direct violations, and censorship pose significant challenges for maintaining academic freedom, conducting the research and education necessary to our profession's integrity, and fostering international academic collaborations in the expansion and dissemination of new knowledge. However, today, academic freedom can no longer be taken for granted. We must constantly discuss, explain, and defend it. As institutions, educators, and scholars, we have a collective responsibility to promote, safeguard, and respect academic freedom. According to Nyirati (2025), we are witnessing a time of deep political and moral division that demands concrete action, not only philosophical discussions. The nursing profession holds a distinctive and crucial place in this struggle, as our ethical duty requires us to reject the misleading “both‐sides” rhetoric that distorts truth in how we generate and share knowledge. Yet, time and time again, we see how the fear of being perceived as “too political” or the instinct to remain neutral fosters a dangerous false equivalence, one that directly conflicts with our nursing ethical obligations (Nyirati 2025). If we step back and allow opinions and ideological values to dictate what and how research is conducted, there is a significant risk that emancipatory and equity‐oriented nursing and health research may become impossible. This means that crucial societal biases and systemic inequalities would remain unchallenged, limiting progress toward more inclusive and effective care.

To ensure that nurses recognize the underlying norms and structures in society that determine who receives care and how it is provided, we must continue to advance critical and emancipatory research within our discipline without fear of censorship or reprisals. Nurses stand on a long and strong legacy of political activism that arises from our moral imperative to actively promote public policy and action that assures social and health equity. Moreover, because healthcare professionals themselves can be carriers of excluding norms in their professional work, we must consciously address them in our education and professional development (Arveklev et al. 2025). We therefore join an international discourse that is becoming increasingly prominent within our discipline, challenging these threats to academic freedom in the name of the nursing profession's ethical foundation.

## Conflicts of Interest

The authors declare no conflicts of interest.

## Data Availability

Data sharing is not applicable to this article as no data sets were generated or analyzed during the current study.

## References

[nin70044-bib-0001] American Nurses Association . 2025. “The Code of Ethics for Nurses.” May 31. https://codeofethics.ana.org/home.

[nin70044-bib-0002] Arendt, H. 2017. The Origins of Totalitarianism. Penguin Books.

[nin70044-bib-0003] Arveklev, S. H. , I. A. Castillo , M. Skalná , et al. 2025. “A Call for Norm‐Consciousness as Points of Departure in Nursing Education.” Nordic Journal of Nursing Research 45: 20571585251332186. 10.1177/20571585251332186.

[nin70044-bib-0004] Carper, B. A. 1978. “Fundamental Patterns of Knowing in Nursing.” Advances in Nursing Science 1, no. 1: 13–24. 10.1097/00012272-19780000-00004. 110216

[nin70044-bib-0005] Castillo, I. A. , E. Tengelin , S. H. Arveklev , and E. Dahlborg . 2024. “When Nursing Education Becomes Political: Norm‐Critical Perspectives in a Campus‐Based Clinical Learning Environment.” Nursing Inquiry 31, no. 2: e12597. 10.1111/nin.12597. 37608629

[nin70044-bib-0006] Chinn, P. 2025. “We Were Made for These Times.” March 19. https://nursology.net/2025/03/19/we-were-made-for-times-like-these/.

[nin70044-bib-0007] Faust, J. 2025. “CDC Orders Mass Retraction and Revision of Submitted Research Across All Science and Medicine Journals. Banned Terms Must Be Scrubbed.” *Inside Medicine*, February 1. https://insidemedicine.substack.com/p/breaking-news-cdc-orders-mass-retraction?r=5p3cr&utm_campaign=post&utm_medium=web&triedRedirect=true.

[nin70044-bib-0008] Flinkfeldt, M. 2025. “Protect Freedom While We Still Have It.” *Curie*, April 7. https://www.tidningencurie.se/kronikor/sla-vakt-om-friheten-medan-vi-annu-har-den.

[nin70044-bib-0009] Fowler, M. 2025. “Banned Words and a Scholarship and Activism of Outrage.” May 28. https://nursology.net/2025/05/28/banned-words-and-a-scholarship-and-activism-of-outrage/.

[nin70044-bib-0010] Georges, J. 2025. “Impending Destruction of NINR?.” April 7. https://nursology.net/2025/04/07/impending-destruction-of-ninr/.

[nin70044-bib-0011] Goldberg, L. , L. Bland , and C. Albright . 2025. “The Impact of American Politics: Canadian Nurses and Our Ethical Mandate.” *Halifax Examiner*, April 14. https://www.halifaxexaminer.ca/commentry/the-impact-of-american-politics-canadian-nurses-and-our-ethical-mandate/.

[nin70044-bib-0012] Kagan, P. N. , M. C. Smith , W. R. Cowling, III , and P. L. Chinn . 2010. “A Nursing Manifesto: An Emancipatory Call for Knowledge Development, Conscience, and Praxis.” Nursing Philosophy 11, no. 1: 67–84. 10.1111/j.1466-769X.2009.00422.x. 20017884

[nin70044-bib-0013] Kalonaityté, V. 2014. Norm‐Critical Pedagogics: For Higher Education. Studentlitteratur.

[nin70044-bib-0014] Kinzelbach, K. , S. Lindberg , L. Lott , and A. Vito Panaro . 2025. “Academic Freedom Index 2025 Update.” Friedrich‐Alexander‐Universität & V‐Dem Institut. https://academic-freedom-index.net/research/Academic_Freedom_Index_Update_2025.pdf.

[nin70044-bib-0015] Liedman, S.‐E. 2020. “Opinions Must Never Be More Important Than Insight.” *Dagens Nyheter*, November 2. https://www.dn.se/kultur/sven-eric-liedman-asikten-far-aldrig-vara-viktigare-an-insikten/.

[nin70044-bib-0016] Lott, L. 2024. “Academic Freedom Growth and Decline Episodes.” Higher Education 88, no. 3: 999–1017. 10.1007/s10734-023-01156-z.

[nin70044-bib-0017] Nye, C. M. , E. Tengelin , and D. Somayaji . 2023. “Developing a Theory of Norm‐Criticism in Nursing Education.” Advances in Nursing Science 46, no. 2: E66–E79. 10.1097/ANS.0000000000000440. 36044350

[nin70044-bib-0018] Nyirati, C. 2025. “False Equivalencies in Nursing Ethics.” March 21. https://nursology.net/2025/03/21/false-equivalencies-in-nursing-ethics/.

[nin70044-bib-0019] Public Health Agency of Sweden . 2025. “Public Health in Sweden: Annual Report 2025.” Folkhälsomyndigheten [In Swedish]. https://www.folkhalsomyndigheten.se/publikationer-och-material/publikationsarkiv/f/folkhalsan-i-sverige-arsrapport-2025/.

[nin70044-bib-0020] Tengelin, E. , E. Dahlborg , I. Berndtsson , and P. H. Bülow . 2020. “From Political Correctness to Reflexivity: A Norm‐Critical Perspective on Nursing Education.” Nursing Inquiry 27, no. 3: e12344. 10.1111/nin.12344. 32009272

[nin70044-bib-0021] Thorne, S. 2025. “We Were Made for These Times.” Nursing Inquiry 32: e70010. 10.1111/nin.70010. 40068129

